# Mechanisms of pathogenesis of emerging adenoviruses

**DOI:** 10.12688/f1000research.10152.1

**Published:** 2017-01-30

**Authors:** James Cook, Jay Radke

**Affiliations:** 1 Division of Infectious Diseases, Department of Medicine, Loyola University Medical Center, 2160 South First Avenue, Maywood, IL 60153, USA

**Keywords:** adenovirus, immunopathogenesis, innate immune response, outbreak

## Abstract

Periodic outbreaks of human adenovirus infections can cause severe illness in people with no known predisposing conditions. The reasons for this increased viral pathogenicity are uncertain. Adenoviruses are constantly undergoing mutation during circulation in the human population, but related phenotypic changes of the viruses are rarely detected because of the infrequency of such outbreaks and the limited biological studies of the emergent strains. Mutations and genetic recombinations have been identified in these new strains. However, the linkage between these genetic changes and increased pathogenicity is poorly understood. It has been observed recently that differences in virus-induced immunopathogenesis can be associated with altered expression of non-mutant viral genes associated with changes in viral modulation of the host innate immune response. Initial small animal studies indicate that these changes in viral gene expression can be associated with enhanced immunopathogenesis *in vivo*. Available evidence suggests the hypothesis that there is a critical threshold of expression of certain viral genes that determines both the sustainability of viral transmission in the human population and the enhancement of immunopathogenesis. Studies of this possibility will require extension of the analysis of outbreak viral strains from a sequencing-based focus to biological studies of relationships between viral gene expression and pathogenic responses. Advances in this area will require increased coordination among public health organizations, diagnostic microbiology laboratories, and research laboratories to identify, catalog, and systematically study differences between prototype and emergent viral strains that explain the increased pathogenicity that can occur during clinical outbreaks.

## Introduction

Human adenoviruses (HAdVs) are non-enveloped, double-stranded DNA viruses of the genus Mastadenovirus in the *Adenoviridae* family. There are seven different species of HAdV (HAdV-A to -G), with over 50 serotypes (numbered consecutively from 1) and 70 distinct genotypes. Different clinical manifestations of infection are, in part, due to the tissue tropism of each species (see Ghebremedhin’s review) ^1^. Thus, HAdVs infect the upper or lower respiratory tracts (species B, C, and E), conjunctiva (species B and D), or gastrointestinal tract (species F and G). Species A–F circulate globally and can cause limited outbreaks of infection. HAdV outbreaks usually occur in closed populations (e.g. hospitals, long-term care facilities, and military installations). Most HAdV infections are diagnosed in children younger than 4 years old. Young children and immunosuppressed adults are more susceptible to HAdVs and are more likely to have more severe infections. HAdV outbreaks might also occur in healthy populations, but since most such infections are self-limited, they are rarely detected by public health surveillance. However, severe pneumonia can occasionally occur and be fatal in immunocompetent patients. The reasons for such severe illness in otherwise healthy patients are unknown.

The existence of over 70 distinct genotypes among the more than 50 serotypes of HAdV suggests ongoing changes in the viral genome as one mechanism of evolutionary diversity. HAdV serotypes are differentiated based on antibody neutralization assays. Genotypes are determined by either restriction endonuclease analysis (REA) or viral genome sequencing. Whereas REA is useful for rapid analysis, it is limited to finding changes in restriction digest patterns. Whole viral genome sequencing combined with bioinformatics provides more precise information on the nature of each new genotype that is discovered. In addition to random mutations, new adenoviral genotypes also result from homologous recombination. Studies by Lukashev *et al.* on clinical isolates of HAdV-C showed that recombination events are common among circulating adenoviruses ^2^. Recombination requires co-infection of the same cell with different viral types. These recombination events usually occur between members of the same species (e.g. HAdV-B with another HAdV-B strain). Interspecies HAdV recombination increases the rate of molecular evolution and results in novel HAdVs that could theoretically have increased viral fitness, altered cell tropism, and increased virulence. However, HAdV recombination usually goes unnoticed because of the self-limited nature of the infections. It is only when mutations or recombinations result in a more pathogenic HAdV genotype that causes outbreaks of more severe disease that the effects of these genetic changes are realized.

## Increased pathogenesis associated with emergent adenovirus outbreaks

There are several examples of outbreaks of respiratory illness resulting in fatalities that have been observed with emerging strains of HAdV. Whereas many cases of severe, HAdV-induced respiratory illness have occurred in patients with underlying and in some cases immunocompromising conditions, there also have been outbreaks of infections among those with no apparent pre-existing risk factors. This review will focus on issues relating to the outbreak viral strains and not an extensive review of possible variations in host defense against infection.

HAdV-3/7 (a recombinant strain) caused an outbreak of acute respiratory infection with fatal outcomes in otherwise healthy infants in Portugal in 2004 ^3^. HAdV-7 has 27 different genotypes that have been associated with fatal disease in immunocompetent patients and tend to be more virulent than other serotypes ^4– 7^. A new genotype of HAdV-14, HAdV-14p1, was identified first in US military populations and then in civilian populations, with outbreaks of respiratory illness of varying severity, including fatal infections ^8– 13^. HAdV-14p1 is now the main HAdV-14 strain circulating worldwide ^14– 19^. Between 2005 and 2013, there were cases of febrile respiratory illness and fatalities in Germany caused by a new genotype of HAdV-21, HAdV-21a ^20^. In 2006 in China, a novel HAdV genotype, HAdV-55, was isolated in outbreaks of severe pneumonia and acute respiratory distress syndrome (ARDS) ^21– 23^. Israel experienced an outbreak of HAdV-55 in 2016 ^24^. HAdV-55 is an intertypic recombinant of HAdV-11 and HAdV-14, with the backbone of HAdV-14 and a partial hexon of HAdV-11 ^25, 26^. When compared with patients with subclinical infections, patients with severe HAdV-55 infections showed significantly higher levels of blood IL-17 ^+^ CD4 ^+^ T lymphocytes and higher levels of serum IFN-γ, IFN-α2, IL-4, and IL-10 ^27^. Why some of these emergent strains induce more intense immune responses and more severe immunopathogenesis in some patients than in others is unclear.

One theory about why these emergent strains cause outbreaks is that the mutations have altered the hexon, fiber, or penton (HAdV structural proteins) that are the targets for neutralizing antibodies, allowing the virus to escape pre-existing herd immunity ^8^. Reduced immunogenicity resulting from changes in viral structural proteins and associated with immune escape has been proposed as an explanation for outbreaks involving emerging strains of group D that cause epidemic keratoconjunctivitis (EKC) ^28^. Some of these emerging EKC strains were observed to induce increased cytokine and chemokine expression and neutrophil infiltrates in a mouse model of conjunctivitis ^28^. In other studies, it was proposed that some emerging EKC strains might be more pro-inflammatory because of the lack of expression of an immunomodulatory RGD domain (integrin ligand) in the viral structural penton base ^29^. Pursuit of such relationships between viral structural protein sequence and function will be required to more completely define their role in increased viral immunopathogenesis.

## Linkage between human adenovirus gene expression and the host immune response

Infection with HAdV turns on a robust innate immune response, resulting from a wide array of viral effects on cellular pathways ^30^. Cellular entry of HAdV triggers strong proinflammatory responses ^31^. The presence of the viral genome induces the cellular inflammasome through the activation of Toll-like receptor signal transduction pathways, resulting in the activation of interferon responses and other proinflammatory cellular defenses ^32– 34^. Viral genome replication induces cell death responses that could kill the host cell before completion of maximal HAdV replication. However, HAdVs have evolved defenses mediated by virally encoded proteins that control the cell death response and repress many of the proinflammatory responses and antiviral effects of host innate immune defender cells that would otherwise limit viral spread, immunopathogenesis, and infection transmission. The functions of such anti-cell-death and immunomodulating HAdV gene products have been best characterized in the HAdV-C species viruses, HAdV-2 and HAdV-5 (see below). Bioinformatics data suggest that the functions of these gene products are similar to those in other HAdVs but not all of these genes are found in every HAdV species.

The adenoviral gene E1A is expressed early after infection, helps drive the cell cycle to increase the efficiency of viral replication, and is necessary for the efficient expression of other viral genes ^35, 36^. E1A also represses both NF-κB-dependent transcription (the major mediator of proinflammatory gene expression) and IFN-stimulated genes, thereby suppressing early inflammatory responses induced by viral entry ^37– 40^.

Products of the *E1B* gene, 19K and 55K, work together to block E1A-induced cell death (apoptosis), and 55K also blocks IFN-stimulated gene expression and other antiviral cellular innate immune reactions ^41– 44^. It would be predicted from these reported immunorepressive effects of E1B 55K that its expression during viral infection would reduce lung inflammation and related cytokine production and that deletion of the *E1B 55K* gene would result in a virus that would cause greater inflammation than wild-type (E1B 55K ^+^) Ad5 during infection. However, the opposite has been reported. Lung infection of either cotton rats (permissive for Ad replication) or mice (nonpermissive) with an *E1B 55K*-deleted Ad5 has been reported to induce less inflammation than infection with wild-type (E1B 55K ^+^) Ad5 and to induce lower levels of lung TNFα and IL-6 ^45^. Our studies revealed that human cells dying from infection with wild-type Ad5 or an *E1B 55K*-deleted Ad5 mutant virus (H5 *dl*338) were highly and equally repressive for macrophage activation responses ^46^. These apparent inconsistencies in the immunoregulatory effects of E1B 55K protein will require further study.

The E3 transcription unit encodes several viral proteins that can alter the innate and adaptive immune responses to HAdV infection ^47^. E3-19K glycoprotein (E3-19Kgp) represses cell surface expression of MHC class I molecules and ligands for the NK cell activating receptor, NKG2D, protecting infected cells from targeting by host killer cells ^48, 49^. There have been varying results of studies of E3-19Kgp expression in determining the host inflammatory response to viral infection. Initial reports indicated that mutant HAdVs lacking the expression of E3-19Kgp induced increased lung inflammatory responses in infection-permissive cotton rats ^50^. Those results suggested that the E3-19Kgp repression of cell surface MHC antigens might reduce inflammation and the generation of virus-specific cytotoxic T lymphocyte (CTL) responses. However, subsequent studies in mice did not find that E3-19Kgp expression during viral infection reduced either virus-specific CTL generation or lung inflammation but suggested that E3B gene products 14.7K, 10.4K, and 14.5K are involved in the repression of the amplitude of the host mononuclear cell inflammatory response in the lung ^51^. The E3 proteins 14.7K, 10.4K, 14.5K, and 6.7K work together to block TNF-induced apoptosis and also play a role in repressing NF-κB-dependent inflammatory responses ^52– 58^. Whereas the *E1A* and *E1B* genes are conserved among HAdV species, the *E3* genes vary markedly. In some strains, one or more of the *E3* genes are absent, and in others there are novel *E3* genes ^59^.

Bioinformatic methods can predict mutations within the more conserved viral genes but might not reveal altered expression of these gene products or the consequences of varying levels of viral gene expression.

## Loss-of-function human adenovirus phenotype associated with increased immunopathogenesis

A novel function of the HAdV-2/5 E1B 19K protein has been described that is associated with the repression of macrophage-mediated inflammatory responses to HAdV-infected cells ^46^. When cells dying as a result of HAdV-5 infection are co-cultured with activated macrophages, the virally infected cells repress pro-inflammatory cytokine production by the macrophages. Deletion of the E1B 19K gene from HAdV-5 results in infected cells that fail to repress and, in the case of some cytokines, enhance the pro-inflammatory responses of activated macrophages. These studies show that, even after HAdV-induced cell death, the virus can still have an impact on the host’s innate immune response. This finding suggested that emergent strains of HAdV that induce increased proinflammatory responses and immune-mediated disease manifestations such as ARDS might have a defect in the expression or function of the viral E1B 19K protein.

HAdV-14p1 has induced global outbreaks for the last 10 years. Sequencing of HAdV-14p1 showed only a 0.3% difference from the prototype HAdV-14 deWit strain, with limited mutations in coding regions ^8^. In nearly all of the HAdV-14p1 outbreaks, there were cases of severe respiratory illness and ARDS that occasionally resulted in death. ARDS causes severe impairment of gas exchange and lung mechanics that are induced by acute lung injury. The inciting event can be a physical injury but in many cases involves either a bacterial or a viral infection. Alveolar macrophages play a key role in both initiating and resolving inflammatory responses associated with ARDS by modulating the expression of IL-1β, IL-6, IL-8, TNF-α, and other pro-inflammatory cytokines ^60^.

**Figure 1.**
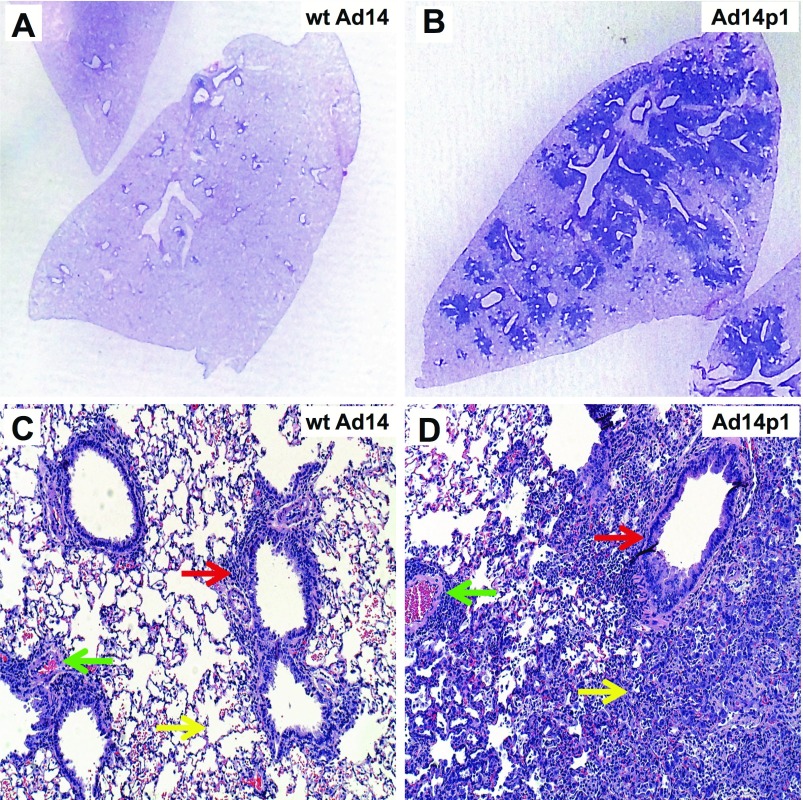
Comparative lung pathology induced by infection with prototype strain Ad14 (wild type, wt) vs. the outbreak strain, Ad14p1. Lungs were harvested from infected hamsters at 7 days after intratracheal viral inoculation (5 × 10 ^9^ plaque-forming units per animal). **A**& **B**, hematoxylin and eosin ( **H**& **E**)-stained lung section macroscopic comparison, showing minimal peribronchial changes in wt Ad14-infected lung ( **A**) vs. marked peribronchial infiltrates in Ad14p1-infected lung ( **B**). **C**& **D**, **H**& **E** comparisons at 10X magnification. Green, yellow, and red arrows indicate perivascular, alveolar, and peribronchial inflammation, respectively, in wt Ad14-infected lung ( **C**) vs. Ad14p1-infected lung ( **D**). Adapted with permission from American Society of Microbiology. Copyright © American Society for Microbiology, [Journal of Virology, volume 90(1), 2016, pages 497-505 and doi: 10.1128/JVI.01790-15].

Studies of the *in vitro* effects of HAdV14-p1 on cultured human cells showed no increase in either cell infectivity or viral replication as compared with the prototype strain ^61^. Lam *et al.* showed that infection of primary human bronchial epithelial cells with HAdV-14p1 resulted in increased expression of IP-10 and I-TAC, two chemokines implicated in ARDS, and speculated that these are potential virulence factors induced by HAdV-14p1 infection ^62^. We observed that HAdV-14p1 infection of human cells results in a marked reduction in E1B 20K expression (the E1B 19K homolog of HAdV-14p1) at the level of transcription, resulting in the formation of larger viral plaques and increased spread of cell pathology in tissue culture compared with the prototype deWit strain of HAdV-14 ^63^. Furthermore, HAdV-14p1-infected human cells either failed to repress or induced increased proinflammatory cytokine expression from human alveolar macrophages, whereas cells dying from infection with the prototype HAdV-14 strain repressed macrophage production of the same cytokines. In a Syrian hamster model of adenoviral pneumonia, HAdV-14p1 induced intense, multifocal inflammatory infiltrates compared with minimal inflammatory responses to the prototype strain ^63^ ( Figure 1). The sequence of the *E1B 20K* gene of HAdV-14p1 was essentially unchanged when compared with the prototype strain (one silent point mutation) ^63^ and would not be predicted to alter *E1B* gene transcription. We postulated, therefore, that one mechanism by which outbreaks of highly virulent HAdV can induce increased lung immunopathology involves loss-of-function phenotypes that result from low-level expression of a normal *E1B 20K* gene product.

## The critical threshold hypothesis of HAdV outbreak strain immunopathogenesis

**Figure 2.**
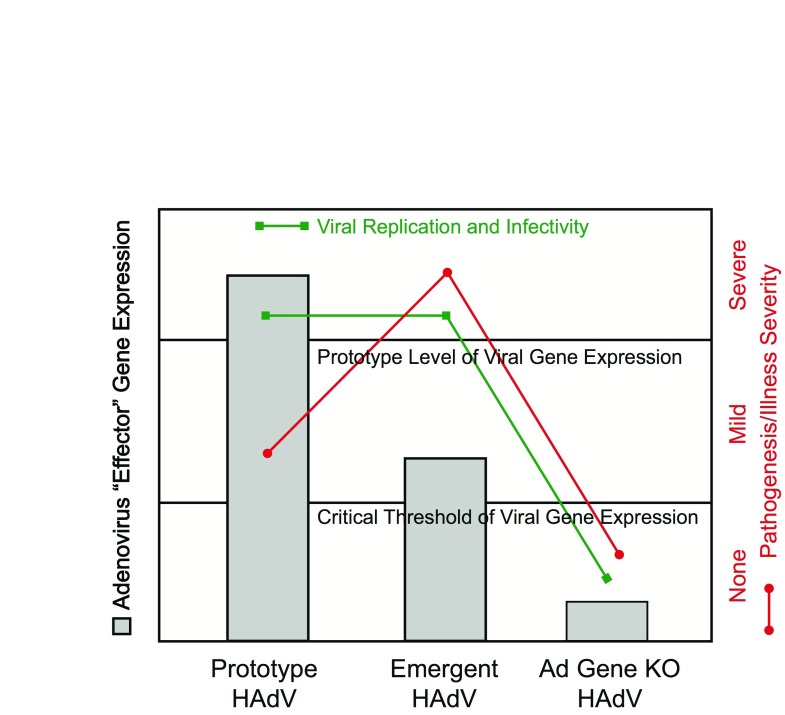
“Critical threshold” viral gene expression model of emergent adenoviral immunopathogenesis. When expression of the adenoviral “effector” gene is normal (left gray bar, Prototype human adenovirus [HAdV]), viral replication and infectivity (green line) are at the rates associated with prototype virus infection, and pathogenesis and illness severity (red line) are mild. Conversely, when viral effector gene expression falls below the critical threshold needed for normal viral replication and infectivity, as would be observed with a gene knockout (KO) virus (right gray bar, Ad Gene KO), there would be inadequate virus generated for infection transmission and little (or no) pathogenesis and no clinical illness. With more virulent, emergent adenoviral strains, expression of the adenoviral effector gene would fall below the normal expression level observed with the prototype strain but would stay above the critical threshold expression level needed for viral replication, infectivity, and infection transmission. Because of the loss-of-function phenotype of the emergent virus that would result from reduced expression of the effector gene, there would be reduced infection-related immunomodulation and, consequently, increased virus-induced immunopathogenesis and more severe clinical illness.

As a result of this observation, we speculate that there is a “critical threshold” level of expression of *E1B 19/20K* that sustains normal Ad14p1 replication and transmission in the human population but fails to convey immunorepressive activity to virally infected cells, resulting in increased proinflammatory cytokine responses and lung injury. This phenotype contrasts with (1) the mild pathogenesis and illness severity associated with the “prototype level” of viral gene expression associated with HAdV-14 (deWit) infection and (2) the reduced infectivity, transmission, and pathogenesis that would occur if the *E1B 19/20K* gene were deleted or rendered nonfunctional by mutation (the equivalent of an *E1B 19/20K* gene knockout) ( Figure 2).

These observations raise the possibility that there might be other evolutionary changes in HAdV gene expression that could fit with the “critical threshold” hypothesis of adenoviral pathogenesis ( Figure 2). Thus, there might be other emergent HAdV strains that induce increased host inflammatory responses (or increases in other parameters of viral pathogenesis) as a result of a reduced expression of otherwise normal viral genes below a critical threshold. In such cases, conventional sequencing studies might not reveal the genetic mechanism(s) of increased viral pathogenesis, since the viral gene that mediates the pathogenic phenotype (the “effector gene”) might be unchanged in sequence but altered in expression because of mutations and functional differences in another viral regulatory gene (the “activator gene”) or sequences (e.g. an enhancer element). It is well established that HAdV encodes key gene products, especially the *E1A* gene family of proteins, that have transcriptional control effects on other viral genes, such as E1B 19/20K ^35^. Therefore, it is theoretically possible that evolutionary changes in the interactions between such viral activator genes or enhancer elements and their targeted viral effector genes could explain the increased pathogenesis of some outbreak strains of HAdV.

## Future studies of the mechanisms of enhanced pathogenesis of outbreak strains of HAdV

Public health surveillance is needed to continue to identify new outbreak strains of HAdV that can cause unusually severe illness. These epidemiological and clinical studies must be coupled with new research approaches in order to understand both the reasons for increased transmission and the mechanisms of increased pathogenicity and virulence of outbreak strains. Sequencing and bioinformatics studies can predict changes in the viral genes and encoded structural proteins – hexon, penton, and fiber – that might result in a change in the sensitivity of the emerging virus to neutralizing antibodies that pre-existed in the population. But formal studies of cross-neutralization of outbreak strains compared with the previously circulating prototype strains, using banks of human sera or virus-induced antisera from animals, would be required to determine whether mutation-related changes in structural protein epitopes could explain the increased transmission of these emerging HAdVs.

Studies will also be needed to more stringently compare the viral “fitness” (i.e. the in-host replication capacity) of the emergent HAdV to their respective prototype strains. It can be assumed that the emergent strains would not succeed in causing outbreaks of infection if they were not at least as fit as the parental strains. However, the question of whether changes in the basics of viral infection, replication, and promulgation have provided any advantage to the outbreak strains that might explain their emergence as a clinically relevant entity would require study of the steps in viral replication, including interactions between viruses and cell surface receptors, viral replication rates, and viral spread from cell to cell.

With the discovery of the immunomodulatory activity of HAdV-infected cells on macrophages, it will be important to continue to develop new *in vitro* model systems to answer questions about how virally infected cells alter the functional activities of other components of the innate and adaptive immune responses to infection. For example, do outbreak HAdV strains that can cause more severe clinical illness induce different virus-infection-related changes in cytokine and chemokine production from other immune cells, such as dendritic cells, T cells, B cells, and NK cells? Do the outbreak strains and the cells they infect alter other immune cell functions such as cellular proliferation, chemotaxis, cytotoxic activity, and antibody production? Answers from these and other *in vitro* studies could provide insights into the possible mechanisms of both the increased success of outbreak strains in avoiding immune surveillance and the increased immunopathogenesis and related organ-specific disease manifestations of infection.

Understanding the translational relevance of *in silico* and *in vitro* studies of comparative changes in parameters of infection and immune response of emergent vs. prototype HAdV strains will require advances in animal model development. Currently, Syrian hamsters and cotton rats are the only virus-replication-permissive small animals available for *in vivo* studies of HAdV infection and pathogenesis. Cotton rats are expensive and difficult to manipulate and maintain in experimental settings. Syrian hamsters are less expensive and easier to use for a variety of studies of viral infection and related pathology. There have been limited hamster-specific reagents available to identify and characterize immune cell types, cytokines, and chemokines at sites of infection and systemically; however, more such reagents are becoming available. Furthermore, the recent description of the use of the CRISPR/Cas-9 system to make a STAT-2 knockout Syrian hamster provides increased options for the use of this animal model for detailed genetic and immunological studies ^64^. It remains to be determined whether HAdV infection of Syrian hamsters will recapitulate the full spectrum of disease manifestations observed in humans and how such animal model studies will correlate with the results of *in vitro* studies using primary human cells.

Not all humans infected during outbreaks of infection with emergent strains of HAdV develop severe illness. Studies of variations in human immune and non-immune responses to outbreak Ad strain infection might be revealing but are limited by the small numbers of people involved and the short durations of these outbreaks. Furthermore, there has been no clear correlation between disease severity and genetic or other predisposing factors of those who have developed severe manifestations of infection. Since viral immunopathogenesis appears to be one determinant of disease severity, studies of differences in the host immune response to infection might provide guidance for future studies of key differences between infections with previously circulating prototype strains and emergent strains of virus at the interface with the host immune response. Progress in developing this understanding will be challenged by the episodic nature of new outbreaks, the relatively small numbers of patients involved in each outbreak, and the limited coordination of such studies at the national and international level.

Coordination between epidemiological studies and laboratory studies of HAdV replication and pathogenesis will require a concerted effort to isolate, identify, genetically characterize, store, and distribute new outbreak strains. The trend in clinical microbiology laboratories is toward the molecular diagnosis of viral respiratory pathogens and away from viral isolation in cell culture. Therefore, emerging diagnostic technology is more likely to result in decreased, not increased, availability of viral isolates for banking, characterizing, and distributing to research laboratories. Since outbreaks of HAdV are usually limited, are not major public health hazards, and are often recognized in hindsight, it is likely that there will continue to be limited resources available to support the public health and laboratory systems needed for advances in HAdV research. These challenges will have to be addressed to make progress in understanding the epidemiology and biology of emerging HAdV infections.

## References

[1] Ghebremedhin B: Human adenovirus: Viral pathogen with increasing importance. *Eur J Microbiol Immunol (Bp).* 2014;4(1):26–33. 10.1556/EuJMI.4.2014.1.2 24678403 PMC3955829

[2] Lukashev AN Ivanova OE Eremeeva TP: Evidence of frequent recombination among human adenoviruses. *J Gen Virol.* 2008;89(Pt 2):380–8. 10.1099/vir.0.83057-0 18198368

[3] Rebelo-de-Andrade H Pereira C Gíria M: Outbreak of acute respiratory infection among infants in Lisbon, Portugal, caused by human adenovirus serotype 3 and a new 7/3 recombinant strain. *J Clin Microbiol.* 2010;48(4):1391–6. 10.1128/JCM.02019-09 20147640 PMC2849616

[4] Zhao S Wan C Ke C: Re-emergent human adenovirus genome type 7d caused an acute respiratory disease outbreak in Southern China after a twenty-one year absence. *Sci Rep.* 2014;4(4): 7365. 10.1038/srep07365 25482188 PMC4258649

[5] Yamamoto D Okamoto M Lupisan S: Impact of human adenovirus serotype 7 in hospitalized children with severe fatal pneumonia in the Philippines. *Jpn J Infect Dis.* 2014;67(2):105–10. 10.7883/yoken.67.105 24647252

[6] Gerber SI Erdman DD Pur SL: Outbreak of adenovirus genome type 7d2 infection in a pediatric chronic-care facility and tertiary-care hospital. *Clin Infect Dis.* 2001;32(5):694–700. 10.1086/319210 11229836

[7] Cui X Wen L Wu Z: Human adenovirus type 7 infection associated with severe and fatal acute lower respiratory illness and nosocomial transmission. *J Clin Microbiol.* 2015;53(2):746–9. 10.1128/JCM.02517-14 25520444 PMC4298532

[8] Houng HS Gong H Kajon AE: Genome sequences of human adenovirus 14 isolates from mild respiratory cases and a fatal pneumonia, isolated during 2006-2007 epidemics in North America. *Respir Res.* 2010;11:116. 10.1186/1465-9921-11-116 20738863 PMC2933684

[9] Louie JK Kajon AE Holodniy M: Severe pneumonia due to adenovirus serotype 14: a new respiratory threat? *Clin Infect Dis.* 2008;46(3):421–5. 10.1086/525261 18173356

[10] Kajon AE Lu X Erdman DD: Molecular epidemiology and brief history of emerging adenovirus 14-associated respiratory disease in the United States. *J Infect Dis.* 2010;202(1):93–103. 10.1086/653083 20500088

[11] Lessa FC Gould PL Pascoe N: Health care transmission of a newly emergent adenovirus serotype in health care personnel at a military hospital in Texas, 2007. *J Infect Dis.* 2009;200(11):1759–65. 10.1086/647987 19842979

[12] Centers for Disease Control and Prevention (CDC): Acute respiratory disease associated with adenovirus serotype 14--four states, 2006-2007. *MMWR Morb Mortal Wkly Rep.* 2007;56(45):1181–4. 18004235

[13] Lewis PF Schmidt MA Lu X: A community-based outbreak of severe respiratory illness caused by human adenovirus serotype 14. *J Infect Dis.* 2009;199(10):1427–34. 10.1086/598521 19351259

[14] Girouard G Garceau R Thibault L: Adenovirus serotype 14 infection, New Brunswick, Canada, 2011. *Emerg Infect Dis.* 2013;19(1):119–22. 10.3201/eid1901.120423 23260201 PMC3557982

[15] Huang S Kamata T Takada Y: Adenovirus interaction with distinct integrins mediates separate events in cell entry and gene delivery to hematopoietic cells. *J Virol.* 1996;70(7):4502–8. 8676475 10.1128/jvi.70.7.4502-4508.1996 PMC190385

[16] Zhang Q Seto D Zhao S: Genome sequence of the first human adenovirus type 14 isolated in China. *J Virol.* 2012;86(12):7019–20. 10.1128/JVI.00814-12 22628402 PMC3393531

[17] Parcell BJ McIntyre PG Yirrell DL: Prison and community outbreak of severe respiratory infection due to adenovirus type 14p1 in Tayside, UK. *J Public Health (Oxf).* 2015;37(1):64–9. 10.1093/pubmed/fdu009 24573364

[18] Carr MJ Kajon AE Lu X: Deaths associated with human adenovirus-14p1 infections, Europe, 2009-2010. *Emerg Infect Dis.* 2011;17(8):1402–8. 10.3201/eid1708.101760 21801616 PMC3381588

[19] O'Flanagan D O'Donnell J Domegan L: First reported cases of human adenovirus serotype 14p1 infection, Ireland, October 2009 to July 2010. *Euro Surveill.* 2011;16(8): pii: 19801. 21371411

[20] Hage E Huzly D Ganzenmueller T: A human adenovirus species B subtype 21a associated with severe pneumonia. *J Infect.* 2014;69(5):490–9. 10.1016/j.jinf.2014.06.015 24975176

[21] Sun B He H Wang Z: Emergent severe acute respiratory distress syndrome caused by adenovirus type 55 in immunocompetent adults in 2013: a prospective observational study. *Crit Care.* 2014;18(4):456. 10.1186/s13054-014-0456-6 25112957 PMC4243941

[22] Li X Kong M Su X: An outbreak of acute respiratory disease in China caused by human adenovirus type B55 in a physical training facility. *Int J Infect Dis.* 2014;28:117–22. 10.1016/j.ijid.2014.06.019 25236387 PMC7128664

[23] Lu QB Tong YG Wo Y: Epidemiology of human adenovirus and molecular characterization of human adenovirus 55 in China, 2009-2012. *Influenza Other Respir Viruses.* 2014;8(3):302–8. 10.1111/irv.12232 24467816 PMC4181478

[24] Salama M Amitai Z Amir N: Outbreak of adenovirus type 55 infection in Israel. *J Clin Virol.* 2016;78:31–5. 10.1016/j.jcv.2016.03.002 26971165

[25] Zhang Q Seto D Cao B: Genome sequence of human adenovirus type 55, a re-emergent acute respiratory disease pathogen in China. *J Virol.* 2012;86(22):12441–2. 10.1128/JVI.02225-12 23087107 PMC3486498

[26] Walsh MP Seto J Jones MS: Computational analysis identifies human adenovirus type 55 as a re-emergent acute respiratory disease pathogen. *J Clin Microbiol.* 2010;48(3):991–3. 10.1128/JCM.01694-09 20042633 PMC2832463

[27] Chen WW Nie WM Xu W: Cross-sectional study of the relationship of peripheral blood cell profiles with severity of infection by adenovirus type 55. *BMC Infect Dis.* 2014;14:147. 10.1186/1471-2334-14-147 24646014 PMC4000060

[28] Walsh MP Chintakuntlawar A Robinson CM: Evidence of molecular evolution driven by recombination events influencing tropism in a novel human adenovirus that causes epidemic keratoconjunctivitis. *PLoS One.* 2009;4(6):e5635. 10.1371/journal.pone.0005635 19492050 PMC2685984

[29] Robinson CM Zhou X Rajaiya J: Predicting the next eye pathogen: analysis of a novel adenovirus. *MBio.* 2013;4(2):e00595–12. 10.1128/mBio.00595-12 23572555 PMC3622935

[30] Hartman ZC Kiang A Everett RS: Adenovirus infection triggers a rapid, MyD88-regulated transcriptome response critical to acute-phase and adaptive immune responses *in vivo*. *J Virol.* 2007;81(4):1796–812. 10.1128/JVI.01936-06 17121790 PMC1797572

[31] Gregory SM Nazir SA Metcalf JP: Implications of the innate immune response to adenovirus and adenoviral vectors. *Future Virol.* 2011;6(3):357–74. 10.2217/fvl.11.6 21738557 PMC3129286

[32] Barlan AU Griffin TM McGuire KA: Adenovirus membrane penetration activates the NLRP3 inflammasome. *J Virol.* 2011;85(1):146–55. 10.1128/JVI.01265-10 20980503 PMC3014182

[33] Barlan AU Danthi P Wiethoff CM: Lysosomal localization and mechanism of membrane penetration influence nonenveloped virus activation of the NLRP3 inflammasome. *Virology.* 2011;412(2):306–14. 10.1016/j.virol.2011.01.019 21315400 PMC3060956

[34] Stein SC Lam E Falck-Pedersen E: Cell-specific regulation of nucleic acid sensor cascades: a controlling interest in the antiviral response. *J Virol.* 2012;86(24):13303–12. 10.1128/JVI.02296-12 23015711 PMC3503059

[35] Berk AJ: Adenovirus promoters and E1A transactivation. *Annu Rev Genet.* 1986;20:45–79. 10.1146/annurev.ge.20.120186.000401 3028247

[36] Frisch SM Mymryk JS: Adenovirus-5 E1A: paradox and paradigm. *Nat Rev Mol Cell Biol.* 2002;3(6):441–52. 10.1038/nrm827 12042766

[37] Cook JL Walker TA Worthen GS: Role of the E1A Rb-binding domain in repression of the NF-kappa B-dependent defense against tumor necrosis factor-alpha. *Proc Natl Acad Sci U S A.* 2002;99(15):9966–71. 10.1073/pnas.162082999 12119420 PMC126608

[38] Ackrill AM Foster GR Laxton CD: Inhibition of the cellular response to interferons by products of the adenovirus type 5 E1A oncogene. *Nucleic Acids Res.* 1991;19(16):4387–93. 10.1093/nar/19.16.4387 1832217 PMC328624

[39] Shao R Hu MC Zhou BP: E1A sensitizes cells to tumor necrosis factor-induced apoptosis through inhibition of IkappaB kinases and nuclear factor kappaB activities. *J Biol Chem.* 1999;274(31):21495–8. 10.1074/jbc.274.31.21495 10419449

[40] Janaswami PM Kalvakolanu DV Zhang Y: Transcriptional repression of interleukin-6 gene by adenoviral E1A proteins. *J Biol Chem.* 1992;267(34):24886–91. 1332971

[41] Sabbatini P Chiou SK Rao L: Modulation of p53-mediated transcriptional repression and apoptosis by the adenovirus E1B 19K protein. *Mol Cell Biol.* 1995;15(2):1060–70. 10.1128/MCB.15.2.1060 7823921 PMC232006

[42] Teodoro JG Branton PE: Regulation of p53-dependent apoptosis, transcriptional repression, and cell transformation by phosphorylation of the 55-kilodalton E1B protein of human adenovirus type 5. *J Virol.* 1997;71(5):3620–7. 9094635 10.1128/jvi.71.5.3620-3627.1997 PMC191510

[43] Chahal JS Gallagher C DeHart CJ: The repression domain of the E1B 55-kilodalton protein participates in countering interferon-induced inhibition of adenovirus replication. *J Virol.* 2013;87(8):4432–44. 10.1128/JVI.03387-12 23388716 PMC3624377

[44] Schreiner S Wimmer P Sirma H: Proteasome-dependent degradation of Daxx by the viral E1B-55K protein in human adenovirus-infected cells. *J Virol.* 2010;84(14):7029–38. 10.1128/JVI.00074-10 20484509 PMC2898266

[45] Ginsberg HS Moldawer LL Prince GA: Role of the type 5 adenovirus gene encoding the early region 1B 55-kDa protein in pulmonary pathogenesis. *Proc Natl Acad Sci U S A.* 1999;96(18):10409–11. 10.1073/pnas.96.18.10409 10468621 PMC17901

[46] Radke JR Grigera F Ucker DS: Adenovirus E1B 19-kilodalton protein modulates innate immunity through apoptotic mimicry. *J Virol.* 2014;88(5):2658–69. 10.1128/JVI.02372-13 24352454 PMC3958100

[47] Burgert HG Ruzsics Z Obermeier S: Subversion of host defense mechanisms by adenoviruses. *Curr Top Microbiol Immunol.* 2002;269:273–318. 10.1007/978-3-642-59421-2_16 12224514

[48] Burgert HG Kvist S: An adenovirus type 2 glycoprotein blocks cell surface expression of human histocompatibility class I antigens. *Cell.* 1985;41(3):987–97. 10.1016/S0092-8674(85)80079-9 3924414

[49] McSharry BP Burgert HG Owen DP: Adenovirus E3/19K promotes evasion of NK cell recognition by intracellular sequestration of the NKG2D ligands major histocompatibility complex class I chain-related proteins A and B. *J Virol.* 2008;82(9):4585–94. 10.1128/JVI.02251-07 18287244 PMC2293069

[50] Ginsberg HS Lundholm-Beauchamp U Horswood RL: Role of early region 3 (E3) in pathogenesis of adenovirus disease. *Proc Natl Acad Sci U S A.* 1989;86(10):3823–7. 10.1073/pnas.86.10.3823 2726753 PMC287233

[51] Sparer TE Tripp RA Dillehay DL: The role of human adenovirus early region 3 proteins (gp19K, 10.4K, 14.5K, and 14.7K) in a murine pneumonia model. *J Virol.* 1996;70(4):2431–9. 8642671 10.1128/jvi.70.4.2431-2439.1996 PMC190086

[52] Shisler J Yang C Walter B: The adenovirus E3-10.4K/14.5K complex mediates loss of cell surface Fas (CD95) and resistance to Fas-induced apoptosis. *J Virol.* 1997;71(11):8299–306. 9343182 10.1128/jvi.71.11.8299-8306.1997 PMC192288

[53] Zilli D Voelkel-Johnson C Skinner T: The adenovirus E3 region 14.7 kDa protein, heat and sodium arsenite inhibit the TNF-induced release of arachidonic acid. *Biochem Biophys Res Commun.* 1992;188(1):177–83. 10.1016/0006-291X(92)92366-6 1417842

[54] Krajcsi P Dimitrov T Hermiston TW: The adenovirus E3-14.7K protein and the E3-10.4K/14.5K complex of proteins, which independently inhibit tumor necrosis factor (TNF)-induced apoptosis, also independently inhibit TNF-induced release of arachidonic acid. *J Virol.* 1996;70(8):4904–13. 8763993 10.1128/jvi.70.8.4904-4913.1996 PMC190440

[55] Friedman JM Horwitz MS: Inhibition of tumor necrosis factor alpha-induced NF-kappa B activation by the adenovirus E3-10.4/14.5K complex. *J Virol.* 2002;76(11):5515–21. 11991979 10.1128/JVI.76.11.5515-5521.2002 PMC137041

[56] Lichtenstein DL Doronin K Toth K: Adenovirus E3-6.7K protein is required in conjunction with the E3-RID protein complex for the internalization and degradation of TRAIL receptor 2. *J Virol.* 2004;78(22):12297–307. 10.1128/JVI.78.22.12297-12307.2004 15507617 PMC525093

[57] Benedict CA Norris PS Prigozy TI: Three adenovirus E3 proteins cooperate to evade apoptosis by tumor necrosis factor-related apoptosis-inducing ligand receptor-1 and -2. *J Biol Chem.* 2001;276(5):3270–8. 10.1074/jbc.M008218200 11050095

[58] McNees AL Gooding LR: Adenoviral inhibitors of apoptotic cell death. *Virus Res.* 2002;88(1–2):87–101. 10.1016/S0168-1702(02)00122-3 12297329

[59] Robinson CM Seto D Jones MS: Molecular evolution of human species D adenoviruses. *Infect Genet Evol.* 2011;11(6):1208–17. 10.1016/j.meegid.2011.04.031 21570490 PMC3139803

[60] Aggarwal NR King LS D'Alessio FR: Diverse macrophage populations mediate acute lung inflammation and resolution. *Am J Physiol Lung Cell Mol Physiol.* 2014;306(8):L709–25. 10.1152/ajplung.00341.2013 24508730 PMC3989724

[61] Anderson BD Barr KL Heil GL: A comparison of viral fitness and virulence between emergent adenovirus 14p1 and prototype adenovirus 14p strains. *J Clin Virol.* 2012;54(3):265–8. 10.1016/j.jcv.2012.03.006 22484030 PMC3367116

[62] Lam E Ramke M Warnecke G: Effective Apical Infection of Differentiated Human Bronchial Epithelial Cells and Induction of Proinflammatory Chemokines by the Highly Pneumotropic Human Adenovirus Type 14p1. *PLoS One.* 2015;10(7):e0131201. 10.1371/journal.pone.0131201 26168049 PMC4500402

[63] Radke JR Yong SL Cook JL: Low-Level Expression of the E1B 20-Kilodalton Protein by Adenovirus 14p1 Enhances Viral Immunopathogenesis. *J Virol.* 2015;90(1):497–505. 10.1128/JVI.01790-15 26491152 PMC4702539

[64] Fan Z Li W Lee SR: Efficient gene targeting in golden Syrian hamsters by the CRISPR/Cas9 system. *PLoS One.* 2014;9(10):e109755. 10.1371/journal.pone.0109755 25299451 PMC4192357

